# Coronary artery spasm: mechanisms, risk factors, and translational strategies for precision management

**DOI:** 10.3389/fcvm.2026.1743355

**Published:** 2026-02-09

**Authors:** Zhihui Kuang, Ranran Kong, Zhonghua Wang, Siying Zuo, Liwei You, Xuan Wang, Xiaoyun Si, Jinfeng Liang

**Affiliations:** 1 School of Clinical Medicine, Guizhou Medical University, Guiyang, China; 2 The Key Laboratory of Myocardial Remodeling Research, The Affiliated Hospital of Guizhou Medical University, Guiyang, Guizhou, China; 3 Department of Cardiology, Nanfang Hospital, Southern Medical University, Guangzhou, China; 4 Department of Cardiology, Chenzhou First People’s Hospital, Chenzhou, China; 5 Department of Cardiovascular Medicine, The Affiliated Hospital of Guizhou Medical University, Guiyang, Guizhou, China

**Keywords:** coronary artery spasm, endothelial dysfunction, genetic polymorphism, perivascular adipose tissue, precision medicine, reactive oxygen species, Rho-kinase, risk factors

## Abstract

Coronary artery spasm (CAS) is a major form of coronary vasomotor dysfunction within ischemia with non-obstructive coronary arteries (INOCA). Despite ethnic and geographic variation in prevalence, CAS is underrecognized because confirmation often requires pharmacological provocation testing that is unavailable in many centers. Clinically, CAS ranges from silent ischemia and angina to acute myocardial infarction, malignant arrhythmias, heart failure, and sudden cardiac death. Evidence suggests that spasm often occurs at sites with mild atherosclerosis and that its risk profile differs from atherosclerotic cardiovascular disease, with more consistent links to smoking, inflammatory burden, and genetic susceptibility, while associations with hypertension and diabetes remain inconsistent. Advances in invasive coronary function testing and recognition of microvascular spasm support an integrated framework involving endothelial dysfunction, vascular smooth muscle hyperreactivity, inflammation–oxidative stress, and autonomic dysregulation. This review synthesizes mechanistic and clinical evidence across these domains, highlights translational opportunities for phenotype-informed risk stratification and precision management, and outlines key research priorities to improve CAS care.

## 1 Introduction

Coronary artery spasm (CAS) is a major form of coronary vasomotor dysfunction and a key endotype within ischemia with non-obstructive coronary arteries (INOCA). Although prevalence varies across ethnic and geographic populations, CAS remains underrecognized because the diagnosis often relies on pharmacological provocation testing that is not routinely performed in many centers. Clinically, CAS ranges from silent myocardial ischemia and angina to acute myocardial infarction, life-threatening ventricular arrhythmias, heart failure, and sudden cardiac death (1).

Early pathological and intravascular imaging studies suggest that CAS frequently occurs at sites with subclinical or mild atherosclerosis, indicating a functional–structural interplay between vasomotor instability and atherosclerotic remodeling (2). Observational comparisons further indicate that the risk factor profile of CAS differs from that of atherosclerotic cardiovascular disease (ASCVD): associations with hypertension and diabetes are inconsistent or weak, whereas smoking, inflammatory burden, and genetic susceptibility show more reproducible links and stronger mechanistic plausibility (3, 4).

In parallel with mechanistic heterogeneity, CAS also exhibits substantial clinical phenotype heterogeneity. CAS can present across distinct ischemic phenotypes, including angina with non-obstructive coronary arteries (ANOCA) and myocardial infarction with non-obstructive coronary arteries (MINOCA). Emerging outcome data suggest worse prognosis in CAS-related MINOCA than in CAS presenting as ANOCA (5), underscoring the need for phenotype-informed risk stratification and management. These observations align with recent calls for precision approaches in vasomotor disorders (6), emphasizing that CAS should not be viewed as a uniform clinical entity.

Systematic synthesis of the risk factors and pathobiological pathways underlying CAS is essential for advancing precision prevention, individualized diagnosis, and targeted therapeutic strategies. To improve interpretability and avoid overstatement, we apply a structured evidence framework throughout this review. We first summarize the pathophysiological mechanisms of CAS, then integrate its risk factor landscape, and finally discuss translational implications and future research priorities.

## 2 Methodological approach of this review

To enhance interpretability and avoid overstatement, we apply a structured evidence framework throughout this review. We distinguish observational clinical studies (cohort, case–control, registry, and case-series data), mechanistic and experimental investigations (cellular, animal, and human physiological studies), and interventional clinical evidence (randomized or non-randomized therapeutic studies). We describe observational findings as associations, mechanistic studies are framed as biological plausibility, and causal language is reserved for interventional evidence or guideline-endorsed recommendations. Where applicable, guideline statements are reported using Class of Recommendation (COR) and Level of Evidence (LOE).

## 3 Microvascular spasm: diagnostic limitations and the epicardial–microvascular continuum

Microvascular spasm is increasingly recognized in vasomotor dysfunction, yet its diagnosis is less straightforward than epicardial spasm because it lacks a direct angiographic correlate. Current definitions rely on acetylcholine-provoked ischemic symptoms and/or ischemic ECG changes without angiographic epicardial constriction (consensus criteria) (7, 8). As a result, diagnostic classification can be sensitive to protocol differences (e.g., acetylcholine dosing), interpretive thresholds, and test–retest/inter-operator variability in symptom and ECG assessment, motivating ongoing standardization of invasive testing (9, 10). Mechanistic and treatment data for microvascular spasm remain largely derived from small physiological or observational studies, whereas epicardial spasm is supported by larger provocation-tested cohorts and more extensive clinical evidence (8, 11, 12).

Emerging invasive data and contemporary syntheses suggest that epicardial and microvascular spasm often overlap and may represent a continuum of coronary vasomotor dysregulation driven by shared upstream pathways [e.g., endothelial dysfunction, Rho-kinase (ROCK) signaling, autonomic imbalance, inflammation] (13, 14). This framing helps interpret heterogeneous results and prevents overgeneralization of therapies across distinct spasm endotypes (10, 13, 14).

CAS can present as ANOCA or MINOCA, and this distinction has pragmatic implications for risk stratification and follow-up. In ANOCA, CAS is often managed as a recurrent symptom disorder, with emphasis on trigger identification, optimization of vasodilator therapy, and relapse prevention, while follow-up is largely guided by symptom control and functional limitation. In MINOCA, spasm-positive testing occurs within an acute coronary syndrome phenotype, where short-to-intermediate event risk and recurrent ischemic injury become more central; closer surveillance and a lower threshold for escalation of preventive strategies may therefore be appropriate (5). Because MINOCA is mechanistically heterogeneous, a spasm finding should be interpreted alongside evaluation for competing or concomitant mechanisms (e.g., plaque disruption or thromboembolism) where clinically feasible, rather than treated as a sole explanation. This phenotype-informed framing aligns with emerging precision approaches in vasomotor disorders (6).

## 4 Pathophysiological mechanisms: the central pathobiological network—an “Amplification Loop” linking endothelial dysfunction to smooth muscle hyperreactivity

**Figure 1:**
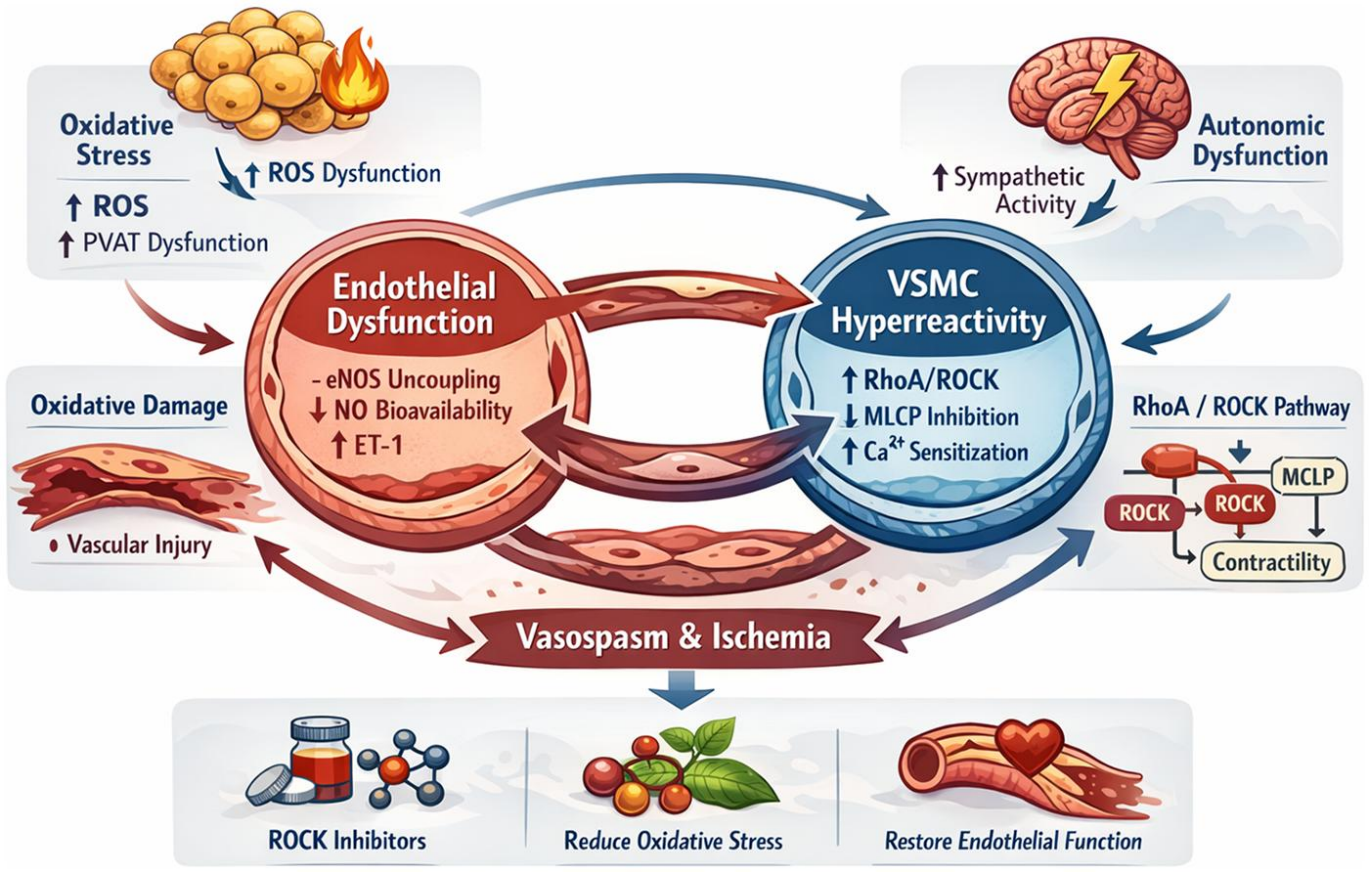
Pathological amplification loop in CAS. This figure illustrates the interconnected molecular and physiological processes that form a self-reinforcing pathological amplification loop in CAS. The loop is centered on endothelial dysfunction and vascular smooth muscle cell (VSMC) hyperreactivity, integrating inflammatory, oxidative, autonomic, and genetic mechanisms. Endothelial injury characterized by eNOS uncoupling, reduced nitric oxide (NO) bioavailability, and increased endothelin-1 (ET-1) expression initiates vasomotor instability. Inflammatory activation and oxidative stress promote perivascular adipose tissue (PVAT) dysfunction, excessive reactive oxygen species (ROS) generation, and propagation of vascular injury. Activation of the RhoA/ROCK signaling cascade enhances VSMC contractility through myosin light chain phosphatase (MLCP) inhibition and calcium sensitization, while autonomic dysregulation provides physiological triggers for spasm onset. Sustained vasospasm induces ischemia, which further aggravates endothelial injury and oxidative stress, thereby maintaining the vicious cycle. This integrated network highlights potential therapeutic targets, including ROCK inhibition, oxidative stress reduction, and restoration of endothelial function, which may disrupt the amplification loop and prevent recurrent spasm. eNOS, endothelial nitric oxide synthase; ROS, reactive oxygen species; ROCK, Rho-associated coiled-coil containing protein kinase; PVAT, perivascular adipose tissue; VSMC, vascular smooth muscle cell; ET-1, endothelin-1; MLCP, myosin light chain phosphatase; NO, nitric oxide.

CAS is best understood as a dynamic, self-reinforcing network rather than a linear sequence of events (Figure 1). This network is anchored by endothelial dysfunction and vascular smooth muscle cell (VSMC) hyperreactivity and is further shaped by inflammation–oxidative stress coupling, autonomic dysregulation, and genetic susceptibility. Perturbation at any node may propagate across the system and lower the threshold for spasm.

### 4.1 Dual core pathways: coordinated activation of endothelial dysfunction and VSMCs hyperreactivity

Endothelial dysfunction is a core component of CAS pathophysiology, driven by reduced nitric oxide (NO) bioavailability and an imbalance between vasodilator and vasoconstrictor influences. Reduced endothelial nitric oxide synthase (eNOS) activity and tetrahydrobiopterin (BH4) can promote eNOS uncoupling, diminishing NO production and increasing superoxide generation. In parallel, elevated vasoconstrictors such as endothelin-1 (ET-1), serotonin, and histamine may further bias vascular tone toward constriction (15).

VSMCs hyperreactivity serves as a major downstream effector of CAS. RhoA/ROCK signaling is strongly implicated, increasing Ca²^+^ sensitization by inhibiting myosin light-chain phosphatase (MLCP) and thereby facilitating myosin light-chain phosphorylation and sustained contraction. Impaired KATP channel function and increased T-type calcium channel activity may further contribute to hyperreactivity in experimental and clinical physiological studies (16). Together, endothelial dysfunction and VSMCs hyperreactivity create a permissive substrate for coronary spasm.

### 4.2 Central amplifier: the inflammation–oxidative stress– perivascular adipose tissue (PVAT) axis

Inflammatory activation can bridge endothelial dysfunction and VSMCs hyperreactivity. Inflammatory mediators such as high-sensitivity C-reactive protein (hs-CRP) and interleukin-6 (IL-6) have been associated with endothelial injury, ROS generation, and RhoA/ROCK activation in mechanistic and translational studies (17). Oxidative stress can further deplete NO bioavailability, promote eNOS uncoupling, and facilitate oxidation of low-density lipoprotein cholesterol (LDL-C) to oxidized LDL (ox-LDL), thereby sustaining an inflammation–oxidative stress feedback loop that may exacerbate endothelial dysfunction and vascular remodeling (18).

Perivascular Adipose Tissue (PVAT) has also emerged as an active paracrine organ that may modulate vascular homeostasis. Under physiological conditions, PVAT exerts anti-contractile effects through adiponectin-related signaling and anti-inflammatory mediators (19). In obesity and related metabolic states, PVAT may shift toward a pro-contractile phenotype, with increased release of mediators such as chemerin, endothelin-1, TNF-α, and oxidative enzymes, alongside reduced adiponectin. PVAT-derived chemerin has been linked to enhanced VSMCs contractility via RhoA/ROCK signaling and to oxidative stress–related endothelial dysfunction (20), whereas reduced adiponectin may weaken vasodilatory and anti-proliferative buffering (21). This shift may contribute to a local milieu favoring vasoconstriction and impaired endothelial reserve (19, 22).

Maging and histopathological studies further suggest associations between PVAT phenotype and vascular reactivity. Quantitative coronary computed tomography angiography (CCTA) analyses report that the PVAT attenuation index correlates with the incidence and severity of vasospasm (23), suggesting that PVAT may act as a metabolic–inflammatory intermediary linking systemic status to local vasomotor instabilit (22).

### 4.3 Triggering regulator*y* axis: bidirectional modulation by the autonomic nervous system and circadian rhythmicity

The autonomic nervous system (ANS) is implicated in CAS, with sympathetic overactivity contributing to coronary spasm. Sympathetic activation via norepinephrine stimulates α-adrenergic receptors on VSMCs, increases intracellular Ca²^+^, and activates RhoA/ROCK signaling, thereby enhancing VSMCs contractility (24, 25).

Under physiological conditions, parasympathetic activation via acetylcholine (ACh) induces endothelium-dependent vasodilation through NO release. In the setting of endothelial dysfunction, however, ACh may paradoxically evoke direct smooth muscle constriction and precipitate spasm. Seminal clinical investigations have shown that intracoronary ACh infusion can reliably provoke spasm in patients with variant angina—a response abolished by atropine—supporting a pathological shift of parasympathetic signaling from vasodilatory to vasoconstrictive dominance (25, 26).

Evidence from ischemic heart disease suggests that vagus nerve stimulation (VNS), via invasive or transcutaneous approaches, may rebalance autonomic tone and modulate inflammatory signaling; however, its efficacy and safety for CAS remain unproven and should be framed as a hypothesis requiring CAS-specific clinical outcome studies (27).

Clinical autonomic monitoring studies have described dynamic pre-spasm shifts. Heart rate variability analyses indicate that, within minutes preceding spontaneous CAS episodes, the high-frequency (HF) component decreases while the low-frequency/high-frequency (LF/HF) ratio increases, a pattern consistent with transient sympathetic surges accompanied by vagal withdrawal (28). Microneurography further supports the presence of heightened sympathetic activity in patients with vasospastic angina, suggesting that sympathetic predominance may represent a chronic predisposing state rather than a purely episodic phenomenon (29).

Interventional pharmacological data provide clinical support for ROCK involvement in provoked coronary hyperreactivity. In small clinical studies, the ROCK inhibitor fasudil attenuated acetylcholine-induced coronary constriction and was accompanied by improvement in ischemic findings, consistent with ROCK acting as a downstream mediator of hypercontractile responses in susceptible vessels (30). Taken together, available data suggest that autonomic perturbations, endothelial dysfunction, and VSMCs hypercontractility interact to lower the threshold for CAS initiation and recurrence.

### 4.4 Amplification via feedback: spasm–induced ischemia as a driver of endothelial injury and dysfunctional vicious cycle

Coronary spasm-induced ischemia may further aggravate endothelial dysfunction and sustain a vicious cycle of recurrent spasm. Preclinical studies and clinical observational data support several interrelated pathways:

(a) Excessive ROS generation. Ischemia–reperfusion injury increases ROS levels and promotes endothelial damage, further depleting NO and worsening endothelial dysfunction (31).
(b) Inflammatory cytokine activation. Ischemic insult can activate pro-inflammatory mediators such as TNF-α and IL-6, which may impair endothelial function and augment VSMCs contractility (32).
(c) Suppression of endothelial repair capacity. Ischemia may reduce endothelial progenitor cell (EPC) mobilization and accelerate endothelial apoptosis, impairing repair capacity (33).

Together, these processes can lower the threshold for recurrent coronary spasm by reinforcing endothelial injury, oxidative stress, and inflammation.

### 4.5 Genetic susceptibility: core molecular loci and pathophysiological mechanisms

Genetic factors contribute to interindividual variability in CAS susceptibility, with reported loci clustering in pathways that regulate NO bioavailability, oxidative stress handling, and vasoconstrictor signaling (genome-wide association studies and candidate-gene analyses). The ALDH2*2 (rs671) missense variant encodes a low-activity aldehyde dehydrogenase, which can increase reactive aldehyde burden and oxidative stress, thereby favoring NO inactivation and vasomotor hyperreactivity (34). This allele is common in East Asian populations but rare in most European populations, a distribution that may partially contribute to ethnic differences in CAS susceptibility (34). Observational studies further suggest gene–environment interplay, with smoking and alcohol exposure amplifying aldehyde-related oxidative stress and associating with higher spasm propensity among carriers (35).

Beyond ALDH2, the eNOS Glu298Asp (rs1799983) polymorphism has been linked to reduced NO signaling and higher vasospasm risk in case–control and cohort studies (36, 37). In East Asian cohorts, RNF213 p.R4810K has been associated with endothelial and smooth muscle stress phenotypes and enrichment among CAS patients, with observational data linking it to adverse ischemic outcomes (38). In some non-Asian populations, EDN1 Lys198Asn (rs5370) has been associated with diffuse epicardial spasm, potentially reflecting enhanced endothelin-1 signaling (39).

Overall, these findings support a genetic contribution to CAS susceptibility, likely through impaired aldehyde detoxification, oxidative stress, disrupted NO signaling, and augmented vasoconstrictor responses.

### 4.6 Central signaling hub: the multilayered regulatory network of ROCK

**Figure 2:**
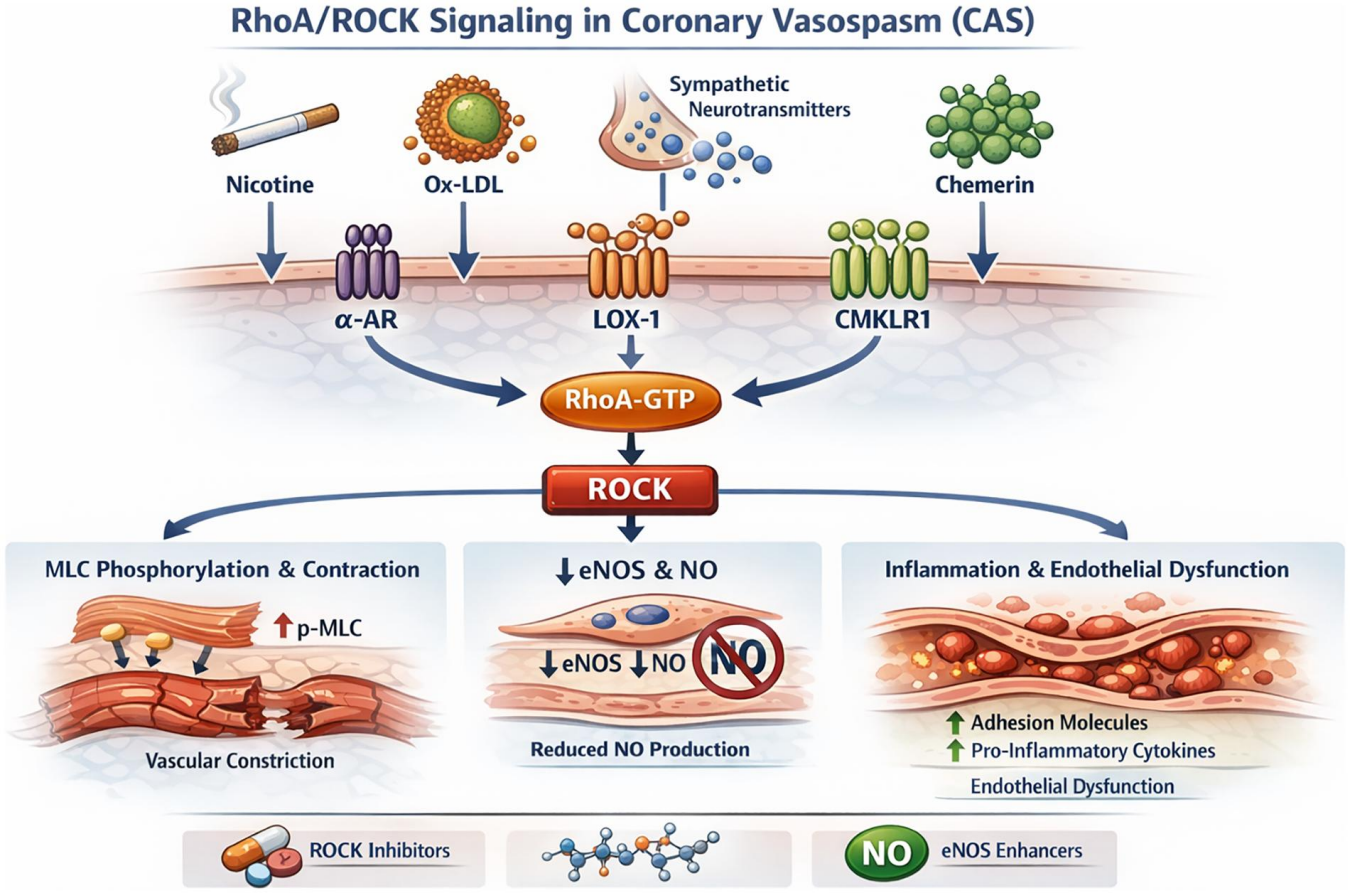
Multidimensional regulatory network of the ROCK signaling pathway in CAS. This schematic depicts the major upstream activators and downstream effectors of the RhoA/ROCK signaling pathway that mediate vascular hypercontractility in CAS. Pathological stimuli, including nicotine, oxidized low-density lipoprotein (ox-LDL), sympathetic neurotransmitters, and chemerin, activate the RhoA/ROCK axis through membrane receptors such as the *α*-adrenergic receptor (*α*-AR), lectin-like oxidized LDL receptor-1 (LOX-1), and CMKLR1. These signals converge to enhance RhoA-GTP binding and ROCK activation. Activated ROCK promotes coronary vasospasm by inhibiting MLCP activity, resulting in sustained myosin light chain (MLC) phosphorylation and increased vascular smooth muscle contraction. In parallel, ROCK suppresses eNOS activity, reduces NO production, and induces pro-inflammatory cytokines and adhesion molecules that perpetuate endothelial dysfunction. This network illustrates how environmental, metabolic, and neurohumoral stimuli converge on ROCK signaling to drive CAS pathophysiology and identifies potential pharmacological checkpoints for intervention. MLCP, myosin light chain phosphatase; LOX-1, lectin-like oxidized LDL receptor-1; CMKLR1, chemerin receptor; α-AR, α-adrenergic receptor; eNOS, endothelial nitric oxide synthase; MLC, myosin light chain; ROCK, Rho-associated coiled-coil containing protein kinase.

As summarized in Figure 2, ROCK can function as a convergent downstream pathway through which diverse upstream triggers translate into enhanced coronary contractility. Experimental and translational studies implicate RhoA/ROCK activation in settings including nicotine exposure, oxidized LDL/lectin-like oxidized LDL receptor-1 (LOX-1) signaling, sympathetic GPCR stimulation, and PVAT-derived adipokines such as chemerin acting via chemerin receptor (CMKLR1) on VSMCs. Once activated, ROCK promotes spasm mainly by increasing Ca²^+^ sensitivity via MLCP inhibition, by impairing endothelial NO signaling through eNOS suppression, and by facilitating a pro-inflammatory milieu that further augments contractile responsiveness. These observations support ROCK as a mechanistically relevant effector and a promising therapeutic target under active investigation in CAS.

## 5 Risk factor landscape

To facilitate critical appraisal, Table 1 summarizes major CAS risk factors, proposed mechanisms, and a structured indication of evidentiary strength (observational, mechanistic, or interventional where available). Ethnic distributions and risk associations of CAS-related genetic polymorphisms are summarized in Table 2.

**Table 1:** Major risk factors for CAS and corresponding evidence strength.

Risk factor category	Specific factor	Core mechanism	Epidemiological evidence	Evidence level	Clinical recommendation	Recommendation strength/notes
Modifiable Lifestyle Factors	Smoking	Smoking induces endothelial dysfunction, increases oxidative stress, and activates the RhoA/ROCK signaling pathway, thereby enhancing vascular smooth muscle contractility.	Meta-analysis (*n* = 9,376): smoking increases MACE risk (RR 1.97, 95% CI: 1.35–2.87) (46); Cross-sectional study (*n* = 275): CAS risk (OR 4.20, 95% CI: 2.93–5.34) (40); Cohort study: stronger association in young/middle-aged STEMI patients (42).	High (observational + guideline-endorsed risk factor)	Complete smoking cessation; integrate into comprehensive cardiac rehabilitation	Guideline-recommended smoking cessation for INOCA risk management (Class I, LOE A) (92, 131)
Modifiable Lifestyle Factors	Dyslipidemia/Atherosclerotic burden	ox-LDL–related endothelial injury, inflammation, ROCK activation, increased VSMCs contractile sensitivity	Cross-sectional study (*n* = 275): CAS risk (OR 2.30, 95% CI: 1.51–3.44) (40); Retrospective cohort (*n* = 80): elevated Lp(a) independently associated with higher spasm activity (48).	Moderate (observational; heterogeneous)	Manage lipids per general dyslipidemia guidelines; intensify LDL-C lowering in high/very-high CV risk	ESC/EAS LDL-C targets (<1.8 mmol/L for high risk; <1.4 mmol/L for very-high risk) rather than CAS-specific targets (130)
Environmental/Drug Factors	Airborne particulate matter (PM2.5/PM10)	Systemic inflammation, endothelin-1/ROCK activation, endothelial dysfunction	Observational study (*n* = 287): long-term PM2.5/PM10 exposure independently associated with CAS in patients with myocardial ischemia without obstructive CAD (NOCAD); stronger association in epicardial spasm and MINOCA (117).	Low–Moderate (observational)	Exposure reduction strategies (avoid high-PM days; respirator masks for high-risk)	Pragmatic risk reduction; not CAS-specific guideline class—state as preventive advice based on observational evidence
Environmental/Drug Factors	Vasoconstrictive drugs (e.g., cocaine; nonselective β-blockers; calcineurin inhibitors)	*α*-adrenergic dominance (cocaine), unopposed α-tone (nonselective β-blockers), endothelial NO impairment (tacrolimus)	Case report: local anesthetic cocaine induced severe CAS (61); Experimental model: cocaine increased coronary vascular resistance six-fold (132); propranolol accentuated cold–induced vasoconstriction (62); atenolol showed no significant CAS increase (133).	Low (case-based/experimental)	Avoid cocaine and triggers; avoid nonselective β-blockers in suspected/known VSA; monitor high-risk exposures	JCS guidance notes concern β-blockers may exacerbate spasm; use caution in high-risk CAS contexts (92)
Anatomical/Comorbid Conditions	MB	Coronary flow turbulence and vessel compression contribute to endothelial dysfunction and abnormal vasomotor regulation.	Cohort (*n* = 310): MB independently predicted MINOCA (OR 2.39, 95% CI: 1.49–3.82); association strongest in ACh-positive cases (67).	Moderate (observational)	Symptom-guided management; consider β_1_-selective blockers for MB-related exertional symptoms	Important nuance: β-blockers can worsen VSA; if CAS coexists, prioritize CCBs/nitrates and individualizeβ_1_-selective use
Anatomical/Comorbid Conditions	OSA	Intermittent hypoxia leads to sympathetic activation, oxidative stress, and endothelial dysfunction.	Case–control (*n* = 62): moderate–severe OSA was associated with a higher odds of CAS (OR 9.61, 95% CI: 2.11–43.78) (122);	Low for CAS-specific; High for CV risk	Screen suspected OSA; treat per sleep medicine standards (CPAP when indicated)	CPAP improves overall CV risk and endothelial function, CAS-specific outcomes remain unproven
Anatomical/Comorbid Conditions	Thyrotoxicosis	Excess thyroid hormone increases myocardial oxygen demand, reduces coronary vasodilator reserve, and heightens catecholamine sensitivity.	Retrospective cohort (*n* = 1,239): hyperthyroidism increases CAS risk 3.27–fold (134); symptoms resolve after thyroid normalization (135).	Low–Moderate (observational)	Prompt thyroid normalization; avoid triggers during thyrotoxic phase	General medical management; not CAS-specific
Non-Modifiable Factors	Age	Endothelial senescence, oxidative stress, reduced NO bioavailability	Retrospective observational study (*n* = 3,155): patients undergoing ACh provocation, the prevalence of CAS increased with age (47.3% for <45 years; 58.3% for 45–54 years; 62.6% for 55–64 years; 61.5% for ≥65 years; *P* < 0.001). Multivariate analysis identified old age as an independent predictor of ACh–induced CAS (adjusted OR 2.60, 95% CI: 2.02–3.24) (82); endothelial dysfunction incidence rises sharply ≥65 years (82, 136).	High (large observational cohorts)	Consider provocation testing based on symptoms/INOCA context rather than age alone	JCS provides indications for provocation testing; avoid presenting age as stand-alone indication (use symptom-driven testing) (92)
Non-Modifiable Factors	Sex	Men: more epicardial spasm (often higher smoking exposure); women: microvascular spasm enriched, post-menopause risk	Gender–stratified analysis (*n* = 104): Korean cohort—majority male with higher smoking/alcohol rates; female patients younger and less exposed (137).	Moderate (observational)	Phenotype-specific evaluation (epicardial vs. microvascular) and risk factor control	Highlight diagnostic heterogeneity
Genetic Variants	ALDH2*2, eNOS Glu298Asp, RNF213, etc.	Aldehyde detox/NO signaling/vascular cell survival pathways	Genomic studies identified ALDH2*2 frequency as markedly higher in East Asians (∼30%–40%) (138); The eNOS Glu298Asp polymorphism conferred approximately a 2.83–fold increase in CAS risk (139); RNF213 p.R4810K variant was associated with a 2.34–fold increased risk (38).	Moderate–High (genetic association)	Not routine screening; consider in strong family history/sudden death or refractory phenotypes	Present as risk modifiers, not direct clinical test recommendations unless local practice supports
Controversial/Inconsistent Factors	Alcohol	In ALDH2*2 carriers: acetaldehyde accumulation, prostanoid imbalance; autonomic effects	Korean cohort (*n* = 5,491): heavy drinking increased CAS risk (HR 1.54, 95% CI: 1.17–2.01) (76); experimental study (*n* = 16): spasm triggered hours post–drinking when plasma ethanol levels approach zero.	Low–Moderate (observational; confounded)	Avoid heavy drinking; consider stricter avoidance in ALDH2*2 carriers	Lifestyle advice; causality uncertain
Controversial Factors	Hyperuricemia	eNOS inhibition, endothelin/ROCK activation	Cohort (*n* = 5,324): no link with overall CAS incidence but multivessel spasm risk was increased by approximately 1.7–fold (99); multivariate analysis confirms uric acid as independent CAS marker (100).	Low–Moderate (observational)	No routine urate-lowering solely for CAS; manage per gout/CKD indications	Marker hypothesis; evidence inconsistent
Controversial Factors	Hypertension	Vascular remodeling; endothelial dysfunction	Cohort (*n* = 938): uncontrolled hypertension associated with 30% lower ACh positivity (3).	Low–Moderate (heterogeneous observational)	Standard blood pressure control per guidelines	No CAS-specific recommendation
Controversial Factors	Glucose metabolism disorders/Diabetes	Fibrosis/endothelial dysfunction; treatment confounding	Cohort (*n* = 986): no significant association between diabetes and CAS (87); insulin resistance prevalent in microvascular dysfunction (140).	Low–Moderate (heterogeneous observational)	Standard glycemic control; assess coronary microvascular dysfunction (CMD) when clinically suspected.	Relevance mainly for microvascular dysfunction

**Footnote (Evidence tier):** High/Moderate/Low reflects overall evidence strength for CAS/VSA relevance: High = consistent multi-cohort/registry support and/or interventional or guideline-backed data; Moderate = heterogeneous observational evidence and/or mechanistic plausibility without outcome validation; Low = small/single-center studies, case series/reports, or hypothesis-generating data with limited replication. Associations are described non-causally unless explicitly supported.

ACh, acetylcholine; CAS, coronary artery spasm; CCB, calcium channel blocker; CI, confidence interval; CMD, coronary microvascular dysfunction; CPAP, continuous positive airway pressure; CV, cardiovascular; hs-CRP, high-sensitivity C-reactive protein; LDL-C, low-density lipoprotein cholesterol; Lp(a), lipoprotein(a); MACE, major adverse cardiovascular events; MB, myocardial bridging; MINOCA, myocardial infarction with non-obstructive coronary arteries; NOCAD, no obstructive coronary artery disease; NO, nitric oxide; OSA, obstructive sleep apnea; OR, odds ratio; ox-LDL, oxidized low-density lipoprotein; PM₂.₅/PM₁₀, particulate matter ≤ 2.5/10 μm; ROCK, Rho-kinase; RR, risk ratio; STEMI, ST-segment elevation myocardial infarction; VSA, vasospastic angina; VSMCs, vascular smooth muscle cells.

**Table 2:** Ethnic differences and risk associations of CAS-related genetic polymorphisms.

Gene variant	Ethnic distribution	Core mechanism	CAS risk	Evidence type	Key references
ALDH2*2	Predominantly East Asian; high carrier frequency in East Asia; impacts alcohol flushing phenotype	Reduced aldehyde dehydrogenase-2 enzymatic activity leads to accumulation of reactive aldehydes, increased oxidative stress, and endothelial dysfunction.	Reported OR 3.00 (95% CI: 1.90–4.80)	Candidate-gene case–control association	JCS VSA guideline (92); Mizuno et al. (141); Rwere et al. (34)
eNOS Glu298Asp	Worldwide distribution.	Reduced endothelial nitric oxide synthase stability and activity, leading to decreased NO bioavailability.	Reported OR 2.83 (95% CI: 1.25–6.41)	Candidate-gene case–control association	Chang et al. (139)
RNF213 p.R4810K	Predominantly East Asian; low carrier rate.	Variant-associated endothelial and vascular smooth muscle cells dysfunction with increased susceptibility to apoptosis.	Reported OR 2.34 (95% CI: 1.99–2.74)	Population-specific variant association (East Asian cohorts)	Hikino et al. (38); Cao et al. (142)
EDN1 rs5370	Multi-ethnic distribution.	Enhanced endothelin-1 gene expression and vasoconstrictor signaling.	Reported OR 1.75 (*P* = 0.009; 95% CI: not reported)	Candidate-gene case–control association	Lee et al. (143)

ALDH2, aldehyde dehydrogenase 2; eNOS, endothelial nitric oxide synthase; RNF213, ring finger protein 213; EDN1, endothelin–1; OR, odds ratio; CI, confidence interval.

### 5.1 Smoking: the most well–defined modifiable risk factor for CAS

Smoking is the most consistently reported modifiable risk factor for CAS. Provocation-tested cohorts, particularly from East Asia, show higher smoking prevalence among CAS patients with dose–response relationships between cumulative exposure and spasm susceptibility (40–42), and smoking is widely regarded as the best-established environmental risk factor (43). Mechanistically, nicotine and combustion-derived oxidants reduce NO bioavailability via oxidative stress, eNOS uncoupling/ADMA-related pathways, and ROCK activation (44–46), and acute exposure may trigger spasm (43). Although CAS-specific cessation trials are lacking, cessation improves vascular function and reduces long-term cardiovascular risk in broader populations (47). it remains a central preventive measure in suspected or confirmed CAS.

### 5.2 Dyslipidemia: the role of oxidative modification in pathophysiology

Conventional dyslipidemia shows heterogeneous and generally modest associations with CAS (40). In contrast, oxidative lipid pathways—ox-LDL, Lp(a), and dysfunctional HDL—are more consistently linked to endothelial dysfunction and VSMCs hyperreactivity (48, 49). LDL oxidation can promote endothelial injury via NADPH oxidase activation and eNOS uncoupling (50), while ox-LDL can amplify contractile signaling through RhoA-related pathways (51). Statins may improve endothelial function and outcomes in selected VSA/CAS populations (52), but evidence for reducing spasm frequency and the role of Lp(a)-targeted strategies remain limited.

### 5.3 Inflammatory burden and hs–CRP: a reproducible association best interpreted as a risk marker

Multiple observational studies report associations between elevated hs–CRP and ACh-induced spasm (3, 53, 54), although effect sizes vary and interactions with sex/metabolic status have been described (55). Mechanistic data support plausibility through impaired NO signaling and ROCK-related facilitation of hyperreactivity (56), and PVAT inflammation may further modulate local vasomotor balance (57). Overall, hs–CRP is best interpreted as a risk marker rather than a uniform causal driver.

### 5.4 Renal-linked biomarkers and systemic vulnerability

In addition to inflammatory markers, renal-linked biomarkers may capture a broader “systemic vulnerability” milieu in which coronary vasomotor dysfunction becomes more likely. Cystatin C has been reported to associate with acetylcholine-provoked CAS in observational cohorts, suggesting that subtle impairment in renal filtration—or the inflammatory, oxidative, and endothelial perturbations that often accompany it—may track with heightened spasm susceptibility (58, 59). Importantly, these data are best interpreted as associative rather than causal: cystatin C integrates multiple upstream processes (renal function, vascular inflammation, and metabolic stress) and therefore remains vulnerable to residual confounding. From a precision-management perspective, its value is less as a standalone discriminator and more as a complementary feature within multimodal risk profiling, helping to contextualize vasomotor testing results and to identify patients whose risk likely reflects a diffuse, multi-organ substrate rather than an isolated coronary phenotype (58, 59).

### 5.5 Drug/toxin exposure: key clinical medication warnings in CAS

Sympathomimetic exposures (e.g., cocaine) can provoke coronary vasoconstriction via adrenergic stimulation and endothelial dysfunction, contributing to ET-1/NO imbalance and ischemic events (60, 61). Non-selective β-blockers may aggravate vasoconstriction through unopposed α-adrenergic signaling (62), whereas β_1_-selective blockers appear less likely to worsen vasospasm (63). Immunosuppressive and chemotherapeutic agents (e.g., tacrolimus, 5-fluorouracil) have been linked to reversible vasospasm during exposure (64, 65). Medication review and avoidance of recognized triggers remain pragmatic measures.

### 5.6 Myocardial bridging: an anatomical substrate associated with spasm susceptibility

Myocardial bridging (MB) is associated with increased spasm susceptibility and contributes to a subset of MINOCA presentations (66, 67). Disturbed shear stress within bridged segments may impair endothelial function (including reduced eNOS expression) and promote local hyperreactivity (68). Provocation testing and intravascular imaging suggest that spasm may localize to or adjacent to bridged segments (67). In MB with unexplained angina or MINOCA, concomitant CAS should be considered, and coronary function testing may be useful where available.

### 5.7 Alcohol intake: a controversial factor with genetic and dose-specific modulation

The relationship between alcohol intake and CAS is heterogeneous. Early reports described alcohol-induced spasm and experimental work suggested vasoconstrictive thresholds (69, 70). Subsequent observations indicate delayed spasm after intake, implicating indirect mechanisms such as endothelial dysfunction and autonomic dysregulation (71, 72). Proposed pathways include altered prostaglandin balance, impaired cGMP-mediated vasodilation, and magnesium depletion affecting eNOS activity (73–76), although these remain hypothesis-generating. ALDH2*2 carriers may be more susceptible to alcohol-related CAS (77). Individualized counseling is appropriate, particularly in patients reporting alcohol-related episodes (78–81).

### 5.8 Age: endothelial senescence and cumulative vascular vulnerability

Advancing age is associated with higher CAS detection in provocation-tested cohorts (82). Endothelial senescence, cumulative oxidative stress, and low-grade inflammation may lower the threshold for vasomotor dysregulation (83–85), and remodeling/subclinical atherosclerosis may further reduce endothelial resilience. These data support considering CAS in older patients with unexplained ischemia, while optimal screening strategies remain uncertain (82, 86).

### 5.9 Glucose metabolism disorders: metabolic dysregulation rather than overt diabetes as a vasomotor modifier

Associations between diabetes and CAS are inconsistent across provocation cohorts (87–89). In contrast, insulin resistance and compensatory hyperinsulinemia may impair NO signaling and microvascular vasodilation, contributing to vasomotor dysregulation even without overt diabetes (88, 90). Follow-up studies also suggest higher incident diabetes in CAS populations (91). Clinically, assessing metabolic status and monitoring for incident diabetes remain appropriate (92, 93).

### 5.10 Hypertension: paradoxical association with CAS susceptibility

Although hypertension contributes to endothelial dysfunction, its relationship with CAS differs from ASCVD. Several provocation-based studies report neutral or inverse associations between hypertension and ACh-induced spasm (3, 94). Proposed mechanisms include remodeling and altered calcium-handling/ROCK signaling that may reduce acute pharmacologic responsiveness in certain contexts (95–98). Hypertension should be treated for overall cardiovascular risk reduction, while its role as a CAS-specific risk factor should be interpreted cautiously.

### 5.11 Hyperuricemia: a marker of spasm severity rather than incidence

Studies on serum uric acid show mixed findings, with inconsistent associations with CAS occurrence but reported links with multivessel spasm in some cohorts (99, 100). Mechanistic studies suggest effects on eNOS, oxidative stress, and endothelin/ROCK pathways (101–103), but CAS-specific interventional confirmation is lacking. Hyperuricemia is therefore best viewed as a potential marker of severity rather than a therapeutic target.

### 5.12 Gender differences and phenotypic heterogeneity

Men more frequently exhibit epicardial spasm, whereas women more often demonstrate microvascular spasm in multicenter observational studies and meta-analyses (104, 105). Estrogen may enhance NO signaling and suppress vasoconstrictor pathways, and post-menopausal changes may contribute to instability (106, 107), although hormone-based interventions remain unproven. Sex-specific interactions with smoking, aging, inflammatory, and metabolic factors have also been reported (54, 108), supporting sex-informed evaluation rather than sex-specific therapy.

### 5.13 Physiological and psychological stress: autonomic dysregulation as a trigger

Autonomic imbalance is frequently observed in vasospastic angina. Heart-rate variability and Holter studies show short-term sympathetic–parasympathetic shifts preceding spasm episodes (109–111). These data are observational and do not establish causality. Where CAS coexists with structural coronary disease, autonomic dysfunction has been associated with worse prognosis (112). Psychological stress may also contribute: mental stress testing can provoke ischemic changes consistent with vasospasm (113), and cohort studies associate anxiety/depression with higher CAS prevalence (114–116), although CAS-specific randomized trials of stress-reduction interventions are lacking.

### 5.14 Environmental factors: external triggers of vasomotor instability

Environmental exposures may act as external triggers that destabilize vasomotor tone in predisposed individuals. Higher long-term PM₂.₅/PM₁₀ exposure has been associated (in multivariable models) with ACh-provocation positivity, with PM₂.₅ showing a stronger relationship with epicardial spasm and both pollutants linked to MINOCA presentations (117). Mechanistic data support plausibility through systemic inflammation, oxidative stress, and downstream ROCK activation, promoting vascular hyperreactivity (118). Cold exposure and hyperventilation are recognized triggers; provocation studies report high sensitivity of combined cold-pressor plus hyperventilation protocols for eliciting variant angina–type responses (119). Overall, the evidence supports an “environmental trigger” model, while causal inference remains limited by exposure assessment and residual confounding.

### 5.15 Ethnicity and genetic susceptibility

Ethnic differences in CAS prevalence are consistently reported, with higher detection rates in East Asian populations than in Western cohorts (104, 120). Genetic studies implicate variants in ALDH2, EDN1, and other vasomotor-related genes as susceptibility loci (39, 77, 121), supporting gene–environment interaction rather than single-gene causation. At present, genetic testing has no established role in routine CAS management.

### 5.16 Comorbid diseases: systemic vasomotor dysregulation

Several comorbid conditions have been associated with CAS, plausibly reflecting systemic vasomotor vulnerability.

**Obstructive sleep apnea (OSA):** Observational studies report higher acetylcholine-provocation positivity in OSA, and CPAP improves endothelial function (122–124). However, CAS-specific interventional trials are unavailable.

**Thyrotoxicosis:** Case reports and small series describe reversible coronary spasm resolving after restoration of euthyroidism (125, 126). Evidence remains largely observational.

**Migraine and Raynaud's Phenomenon:** Both represent vasomotor hyperreactivity phenotypes, and observational studies report higher prevalence among CAS patients (127–129). Shared genetic predisposition has been suggested.

Collectively, these comorbidities are best viewed as markers of systemic vasomotor susceptibility rather than direct causal factors.

## 6 Summary and future directions

CAS is a multifactorial vasomotor disorder in which endothelial dysfunction and VSMCs hyperreactivity interact with inflammation–oxidative pathways, autonomic imbalance, and genetic susceptibility. While parts of the risk profile overlap with ASCVD (e.g., smoking and dyslipidemia), CAS also has distinct modifiers, including myocardial bridging, PVAT-related dysfunction, and population-specific genetic variants that may shape susceptibility and clinical expression.

## 7 Key strategies to improve prognosis include

### 7.1 Risk-factor modification

Smoking cessation remains the most actionable preventive intervention, alongside mitigation of inflammatory burden, avoidance of relevant triggers (including medications and environmental exposures), and optimization of comorbidities such as OSA.

### 7.2 Phenotype-informed therapy and evidence gaps

Calcium channel blockers remain first-line therapy. For refractory cases, small interventional studies suggest that ROCK inhibition (e.g., fasudil) can attenuate ACh-induced constriction and ischemic changes, including in microvascular spasm phenotypes (30, 130). However, robust CAS-specific outcome trials—particularly phenotype-stratified comparisons between epicardial and microvascular spasm—remain limited. Therefore, phenotype-guided pharmacotherapy should be presented as a rational but still unvalidated strategy that requires prospective confirmation.

### 7.3 Priorities for future research

(1) Define how major modifiers (e.g., ALDH2*2, tobacco exposure, PM₂.₅) interact within the “amplification loop,” and identify tractable regulatory nodes.
(2) Determine whether multimodal tools (e.g., leukocyte ROCK activity, PVAT attenuation, genetic risk scores, renal-linked biomarkers) improve risk stratification and treatment selection beyond clinical phenotyping alone; cystatin C associations require external validation (58, 59).
(3) Clarify endotype overlap and diagnostic uncertainty across epicardial and microvascular spasm, and establish standardized, reproducible testing thresholds to reduce misclassification and inappropriate therapeutic generalization.
